# Effect of a Novel Adenosine A2a Receptor Antagonist MB204 on Tau Hyperhosphorylation In Vivo

**DOI:** 10.1002/alz70859_103170

**Published:** 2025-12-25

**Authors:** Mark Stephen Williams, Sofia Diego‐Diaz, Geoffrey Canet, Francis Laliberté, Emmanuel Planel

**Affiliations:** ^1^ Marvel Biosciences, Calgary, AB Canada; ^2^ Laval University, Quebec, QC Canada; ^3^ Université Laval ‐ Centre de Recherche du CHU de Québec, Québec, QC Canada; ^4^ CHU de Quebec, Quebec, QC Canada

## Abstract

**Background:**

Phosphorylation of Tau is a key post‐translational modification implicated in tauopathies, such as Alzheimer’s disease (AD), leading to microtubule dysfunction and Tau aggregation. Recent preclinical evidence suggests that deletion or pharmacological inhibition of the adenosine A2A receptor improves outcomes in both amyloid and Tau models. We recently developed a novel fluorinated derivative of the approved A2A receptor antagonist, Istradefylline (approved for Parkinson’s disease), named MB204, for potential use in AD and depression. Here, we investigate the acute effects of oral MB204 on Tau phosphorylation in a non‐transgenic mouse model of Tau hyperphosphorylation.

**Method:**

Eight‐week‐old CD‐1 male mice (n=6 per group) were dosed with MB204 (2.5 mg/kg, p.o.) or the positive control lithium chloride (LiCl, 25.6 mg/kg, i.p.) daily for three days. One hour after the final dose, general anesthesia (1.3% isoflurane) was administered to lower body temperature and induce Tau hyperphosphorylation, as described previously. Brain tissue was collected 3 hours after anesthesia administration for quantitative western blot analysis targeting multiple Tau phosphorylation epitopes.

**Result:**

Multiple Tau epitopes were studied. In the cortex: reductions were seen in the phosphorylation at AT270, AT8 were seen in both MB204 and Li treatment (both p<0.001). A reduction was only seen with MB204 for pT205 (p<0.001) and only seen with lithium for the pS409 epitope (p<0.001). In the hippocampus: reductions were seen in the phosphorylation at pS40K were seen in both MB204 and Li treatment (both p<0.05 and 0.001 respectively). A reduction was only seen with MB204 for the AT8 epitope (p<0.05) and only seen for lithium for the AT270 and pT205 epitope (p<0.01 and 0.001)

**Conclusion:**

Acute oral treatment of mice with MB204 reduced anesthesia induced Tau hyperphosphorylation at key clinically relevant Tau epitopes. These findings highlight MB204 potential as a therapeutic agent for AD and related tauopathies.

